# Fracture Resistance of CAD/CAM Resin-Matrix Ceramic Overlays and Full-Coverage Crowns for Maxillary Premolars

**DOI:** 10.3390/biomimetics11050291

**Published:** 2026-04-22

**Authors:** Ali Abulkasim Mohamed, Brian Morrow, Stella Mireles, Carlos A. Jurado, Mark A. Antal, Silvia Rojas-Rueda, Hamid Nurrohman, Franklin Garcia-Godoy

**Affiliations:** 1 Division of Operative Dentistry, Department of General Dentistry, College of Dentistry, University of Tennessee Health Science Center, Memphis, TN 38103, USA; 2 Department of Bioscience Research, College of Dentistry, The University of Tennessee Health Science Center, Memphis, TN 38163, USA; 3 College of Dentistry, University of Tennessee Health Science Center, Memphis, TN 38103, USA; 4 School of Dental Medicine, Ponce Health Sciences University, Ponce 00732, Puerto Rico; 5 Department of Operative and Esthetic Dentistry, Faculty of Dentistry, University of Szeged, 6720 Szeged, Hungary; antal.mark@szte.hu; 6 Division of Dental Biomaterials, Department of Clinical and Community Sciences, School of Dentistry, University of Alabama at Birmingham, Birmingham, AL 35233, USA; 7 Department of Restorative Dentistry & Prosthodontics, The University of Texas School of Dentistry, Houston, TX 77054, USA

**Keywords:** partial crowns, CAD-CAM, resin-matrix ceramic, overlay, crowns, premolar

## Abstract

Objective: CAD-CAM technology enables biomimetic dentistry by producing highly accurate, minimally invasive restorations that replicate the biomechanical behavior of intact teeth. This study evaluated the fracture resistance of overlays with margins at different supragingival levels, including a flat occlusal design and compared them with conventional full crowns. All restorations were fabricated from chairside CAD/CAM resin-matrix ceramic for maxillary premolars. Methods and Materials: Sixty-four CAD/CAM resin-matrix ceramic restorations were fabricated and randomly assigned to four groups (n = 16): (1) overlay with a margin 2 mm above the gingiva (Ov2m); (2) overlay with a 4 mm supragingival margin (Ov4m); (3) overlay with a 4 mm margin and flat occlusal surface (OvF4m); and (4) full-coverage crown with a gingival-level margin (FCC). Preparations were standardized by one operator. Restorations were adhesively cemented to resin dies, thermocycled 10,000 times (5–55 °C), and loaded to failure in a universal testing machine (1 mm/min). Data were analyzed using one-way ANOVA and post hoc tests (α = 0.001). Results: Among overlays, Ov2m showed the highest fracture resistance (1605 ± 88 N), followed by Ov4m (1403 ± 63 N). OvF4m recorded the lowest value (1257 ± 73 N). FCC exhibited the greatest overall resistance (1838 ± 106 N), significantly higher than that of any overlay group. Conclusions: Overlays with margins 2 mm above the gingiva had higher fracture resistance than those with more coronal margins or flat occlusal designs. Full-coverage crowns showed the greatest strength, highlighting the impact of margin position and preparation design on restoration performance.

## 1. Introduction

The development of computer-aided design and computer-aided manufacturing (CAD/CAM) systems has provided the high level of precision necessary to fulfill biomimetic restorative objectives in modern dentistry [1,2]. Through the integration of digital design, precise machining, and advanced adhesive protocols, these systems support a minimally invasive philosophy centered on the preservation of healthy tooth structure while restoring the tooth’s structural integrity, function, and esthetics. Initially, CAD/CAM systems were primarily restricted to the fabrication of small, single-unit restorations. Nevertheless, ongoing advances in software, hardware, scanning technology, and material processing have substantially broadened their range of indications. These technologies can now be applied not only to the design and manufacture of single restorations, but also to multiple-unit and more complex prosthetic rehabilitations, in addition to numerous other dental devices [3,4,5]. CAD/CAM-fabricated restorations are widely recognized as clinically reliable and, in many circumstances, can be produced more efficiently and in less time than restorations fabricated through conventional laboratory methods [6,7]. This improved efficiency may also simplify clinical workflows and enhance treatment delivery. Furthermore, studies have shown that patients often prefer digital workflows, especially intraoral scanning, over traditional impression procedures, likely because these techniques are generally more comfortable, less cumbersome, and better accepted from the patient’s perspective [8]. Therefore, CAD/CAM technology has become a valuable tool in restorative dentistry, contributing to greater precision, improved workflow efficiency, enhanced patient comfort, and the advancement of conservative restorative strategies. Ceramic materials compatible with CAD/CAM systems have likewise gained considerable popularity in recent years, mainly because of their excellent esthetic properties and favorable mechanical performance [9,10]. Initially, conventional ceramic materials such as porcelain, leucite-reinforced ceramics, lithium disilicate, and zirconia were adapted for CAD/CAM fabrication. Polymer composites used in dentistry have also demonstrated several advantageous properties. They possess favorable optical characteristics and, when properly manipulated, can achieve an appearance similar to that of natural dentition. Furthermore, they are well aligned with the principles of conservative dentistry because they can be bonded directly to enamel and dentin, thereby requiring less removal of sound tooth structure than traditional restorative approaches. They also exhibit good insulating properties, which may contribute to reduced tooth sensitivity. In addition, their ease of repair is a significant clinical advantage, as they can often be repaired intraorally without requiring complete replacement of the restoration [11,12].

As the field evolved, manufacturers began to introduce hybrid materials that combine ceramics with polymers or with other ceramic components in an effort to optimize overall performance [13,14,15]. The rationale behind these newer formulations is to merge the advantages of different constituents and thereby produce materials with improved properties that may not be achievable with a single-phase material alone. A representative example is polymer-infiltrated feldspathic ceramic, which combines the desirable optical characteristics of feldspathic ceramic with the manufacturing efficiency and simplified processing associated with polymer-containing materials. This combination may allow for faster production and easier handling while maintaining favorable esthetic outcomes, and it may also eliminate additional processing steps required by some conventional ceramic systems [16].

Minimally invasive concepts in restorative dentistry aim to conserve as much natural tooth tissue as possible to support long-term survival of the dentition [17]. Increasingly, clinical reports describe dentists opting for conservative designs rather than traditional cavity or crown preparations [18,19]. For crowns, conservative preparations often feature supragingival margins, instead of the conventional placement at the gingival margin [20,21]. Other innovative proposals have included designs with flat occlusal surfaces [22].

Although some studies have investigated the mechanical performance of conservative, non-traditional restorations, evidence regarding the behavior of CAD/CAM hybrid ceramic designs remains limited. Collecting this information is essential for guiding clinicians in selecting suitable preparation designs for this class of materials. Therefore, this research aimed to assess the fracture resistance of several onlay designs compared with conventional crowns, all fabricated from chairside CAD/CAM resin-matrix ceramic. The tested designs included restorations with finish lines positioned 2 mm or 4 mm above the gingival margin, as well as an onlay with a 4 mm supragingival margin and a flat occlusal surface. The first null hypothesis was that no significant difference in fracture resistance would be found between the onlay groups and the full crown group. The second null hypothesis assumed there would be no difference among the three onlay designs tested in this study.

## 2. Materials and Methods

### 2.1. Specimen Preparation

Four maxillary right first-premolar typodont teeth (1560 Dentoform, Columbia Dentoform, Lancaster, PA, USA) were divided into four experimental groups as follows: (Group 1) overlay with margin 2 mm above the gingival level (Ov.2m); (Group 2) overlay with margin 4 mm above the gingival level (Ov.4m); (Group 3) overlay with margin 4 mm above combined with a flat occlusal surface (OvF.4m); and (Group 4) a full-coverage crown with margin at the gingival level (FCC). All preparations were performed by one experienced prosthodontist. For Groups 1, 2, and 4, the occlusal reduction was standardized to 1.5 mm, while Group 3 was prepared to maintain 1.5 mm at the central occlusal surface. The typodont teeth were scanned using an intraoral chairside CAD/CAM system (Primescan, Dentsply Sirona, Charlotte, NC, USA), and the restorations were digitally designed (CEREC, Dentsply Sirona, Charlotte, NC, USA) to produce standardized contours. A total of 64 restorations (n = 16 per group) were milled with MCXL (Dentsply Sirona, Charlotte, NC, USA) from a CAD/CAM resin-matrix ceramic (Cerasmart, GC, Tokyo, Japan). A priori power analysis for one-way ANOVA (fixed effects, omnibus) was conducted in G*Power 3.1 (α = 0.05, power = 0.80, f = 0.50). The required sample size was 48 specimens (12 per group). To improve robustness and account for variability, 16 specimens per group were included, yielding a total of 64 specimens.

**Figure 1:**
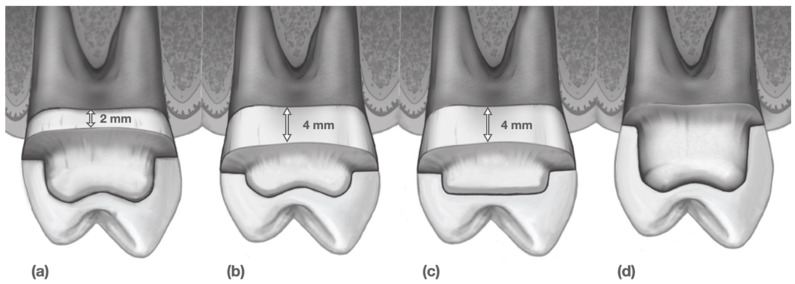
Cross-sectional schematic of the restoration configurations: (**a**) overlay with margin 2 mm above the gingival level (Group 1); (**b**) overlay with margin 4 mm above the gingival level (Group 2); (**c**) overlay with margin 4 mm above the gingival level plus a flat occlusal surface (Group 3); (**d**) full-coverage crown with margin at the gingival level (Group 4).

The ceramic restorations were polished using an all-ceramic polishing kit (Universal Polishing System, Brasseler USA, Savannah, GA, USA). The four prepared typodont teeth were then scanned with a laboratory scanner (Degree of Freedom HD, DOF, Seoul, Republic of Korea), and four dies were digitally created to match the contours of the preparations (Figure 1).

A total of 64 dies (n = 16 per group) were produced from 3D resin (Model Resin, Formlabs, Somerville, MA, USA), which had a tensile strength of 61.0 MPa, a value within the reported range for natural dentin (44.4 to 97.8 MPa) [23,24] using a laboratory printer (FormLab 2, Formlabs, Somerville, MA, USA), a device that has been reported in previous studies [25,26,27]. The intaglio surface of each restoration was cleaned with a specialized paste (Ivoclean, Ivoclar Group, Schaan, Liechtenstein). Printed dies were etched with 39% phosphoric acid (Total Etch, Ivoclar Group, Schaan, Liechtenstein) for 15 s, rinsed, and air-dried. Each restoration was bonded to its respective die using resin luting cement (Multilink Automix, Ivoclar Group, Schaan, Liechtenstein). Light curing was performed for 20 s on mesial, distal, buccal, lingual, and occlusal surfaces at a distance of 5 mm, followed by an additional 20 s of self-curing under a constant 200 g load. The bonded specimens were stored in water at room temperature until mechanical testing.

### 2.2. Fracture Testing Procedure

All specimens were subjected to artificial aging in a thermocycling machine (Sabri Dental Enterprises Inc., Downers Grove, IL, USA). A total of 10,000 cycles was performed between two water baths, one maintained at 5 °C and the other at 55 °C. Each cycle lasted 60 s, consisting of 20 s in the cold bath, 10 s for transfer, 20 s in the hot bath, and another 10 s to return the samples to the cold bath. The restorations were then positioned in a jig to apply vertical loading at the occlusal surface. A 1 mm plastic sheet was placed between the occlusal surface and the flat-shaped applicator to simulate food bolus and distribute the forces uniformly. A universal testing machine (ElectroPuls E3000, Instron, Norwood, MA, USA) was operated at a crosshead speed of 1 mm/min covering the entire occlusal surface and the load was applied vertically until fracture occurred. The fracture load was measured in Newtons for every specimen in each group.

### 2.3. Scanning Electron Microscope Observation

The fractured surfaces of the restorations were examined using field emission scanning electron microscopy (SEM) (EVO HD15, Zeiss, Oberkochen, Germany). The specimens were coated with a thin gold layer of about 100 Å using a sputter coater (Desk II, Denton Vacuum, Moorestown, NJ, USA) to provide conductivity. Images were obtained at an accelerating voltage of 5 kV.

### 2.4. Statistical Analysis

Fracture load values were reported as mean, standard deviation, and range. Statistical evaluation of fracture strength was performed using a commercial software package (SPSS Statistics 25, IBM, Armonk, NY, USA). A one-way analysis of variance (ANOVA) followed by Tukey post hoc testing was conducted, with statistical significance set at α = 0.05.

## 3. Results

### 3.1. Fracture Testing Results

Table 1 summarizes the fracture load values obtained for overlays with different designs and for conventional full-coverage crowns fabricated from CAD/CAM resin-matrix ceramic in maxillary premolars. Restoration design had a significant effect on fracture strength (F(3,60) = 143.118, *p* < 0.001). Among the overlay groups, specimens in Group 1 (Ov.2m), with margins positioned 2 mm above the gingival level, exhibited the highest resistance (1605 N). Group 2 (Ov.4m), with margins 4 mm above the gingival level, showed lower values (1403 N), while Group 3 (OvF.4m), with a flat occlusal surface and 4 mm supragingival margin, demonstrated the lowest resistance (1257 N). In contrast, Group 4 (FCC), the conventional full-coverage crowns with margins at the gingival level, presented the highest overall fracture load (1838 N), surpassing all overlay configurations. One-way ANOVA confirmed that the differences among groups were statistically significant. Tukey post hoc analysis showed that all pairwise comparisons were statistically significant (*p* < 0.001).

### 3.2. Scanning Electron Microscope (SEM) Observations

**Figure 2:**
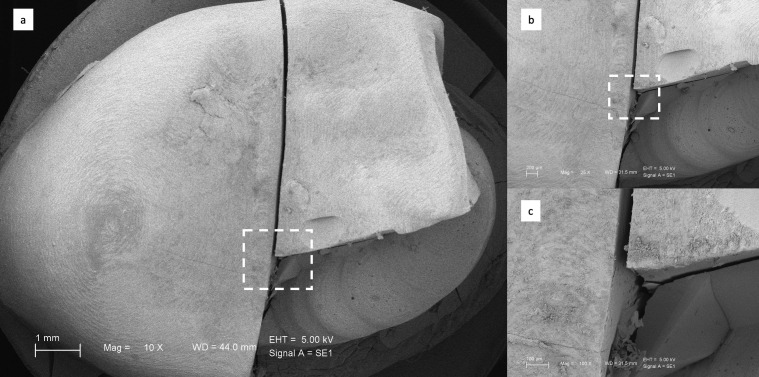
Representative SEM views of Group 1 overlays at (**a**) ×10, (**b**) ×25, and (**c**) ×100 magnifications.

**Figure 3:**
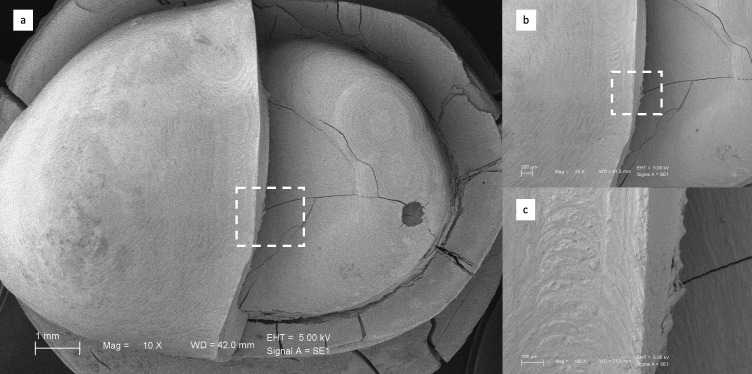
Representative SEM views of Group 2 overlays at (**a**) ×10, (**b**) ×25, and (**c**) ×100 magnifications.

**Figure 4:**
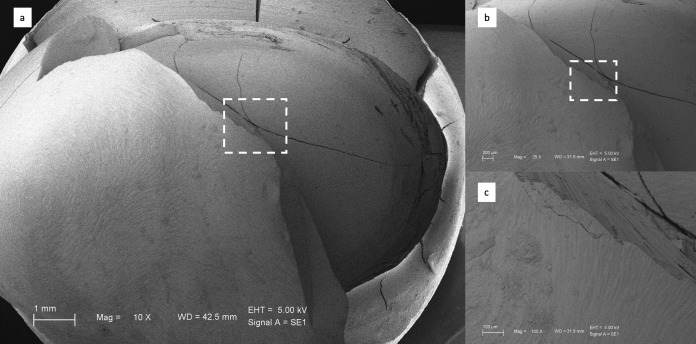
Representative SEM views of Group 2 overlays at (**a**) ×10, (**b**) ×25, and (**c**) ×100 magnifications.

**Figure 5:**
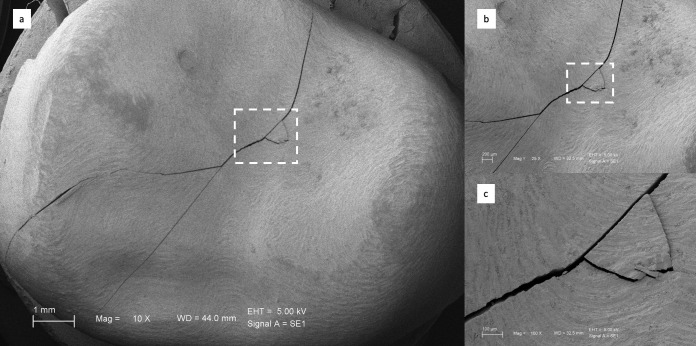
Representative SEM images of Group 4 full crowns at (**a**) ×10, (**b**) ×25, and (**c**) ×100 magnifications.

Figure 2, Figure 3, Figure 4 and Figure 5 present representative SEM micrographs of fractured specimens at 10×, 25×, and 100× magnifications. The fractured surfaces were analyzed fractographically. Group 4 full-coverage crowns (FCCs) exhibited fewer and less pronounced crack lines, whereas all overlay designs showed a greater number of cracks with irregular patterns. Within the overlay groups, specimens from Group 3 (OvF.4m), which had the lowest fracture resistance, also displayed the highest number and most irregular cracks. By comparison, overlays from Group 1 (Ov.2m) and Group 2 (Ov.4m) demonstrated smoother fracture lines with cleaner patterns.

## 4. Discussion

Minimally invasive dentistry, grounded in biomimetic principles, emphasizes the preservation of healthy dental tissue while restoring the tooth’s natural structural integrity and functional performance. This philosophy has promoted the development of partial-coverage restorations as conservative alternatives to conventional full-coverage crowns. In the present in vitro study, the fracture resistance of chairside CAD/CAM overlays with different preparation designs was compared with that of conventional full crowns fabricated from a resin-matrix ceramic material in maxillary premolars. The first null hypothesis, which proposed that no differences would exist between the overlay designs and the traditional crown design, was rejected. The results demonstrated clear differences among the groups. Group 1 showed a fracture resistance of 1605.69 N, Group 2 reached 1403.81 N, and Group 3 recorded 1257.38 N, whereas Group 4, representing the traditional full-crown design, exhibited the highest value at 1838.00 N. These findings indicate that restoration design had a substantial influence on fracture resistance. The second null hypothesis, which stated that no differences would be found among the three overlay groups, was also rejected, as statistically significant differences were identified among Groups 1, 2, and 3. Therefore, both the type of restoration and the specific preparation design appear to play important roles in the mechanical behavior of CAD/CAM restorations under loading conditions.

Chairside CAD/CAM approaches have become increasingly popular among clinicians because they allow restorations to be designed, fabricated, and delivered in less time than conventional techniques [28,29,30]. This reduction in treatment time is one of the major advantages of digital dentistry, particularly in clinical settings where efficiency and patient convenience are important considerations. One randomized controlled trial compared digital and conventional workflows for the fabrication of lithium disilicate crowns in 10 participants and found that laboratory working time was consistently shorter when digital methods were used, regardless of the CAD/CAM system selected [31]. These findings support the view that digital workflows can streamline the restorative process without compromising clinical feasibility. In agreement with this, a recent systematic review that evaluated studies published between 2010 and 2023, including eight investigations, confirmed that digital methods significantly reduce processing time when compared with conventional approaches [32]. Taken together, these reports reinforce the growing preference for chairside CAD/CAM systems in restorative dentistry. Considering their demonstrated time efficiency, clinical practicality, and widespread use in contemporary practice, a chairside CAD/CAM workflow was selected for the fabrication of the restorations evaluated in the present study.

CAD/CAM hybrid materials, particularly resin-matrix ceramics, have gained wide acceptance in restorative dentistry because of their favorable clinical performance [33,34,35]. A systematic review of partial-coverage restorations published between 2005 and 2020 reported success rates ranging from 85.7% to 100% for inlays, onlays, and overlays, highlighting the reliability of these treatment options [36]. Similarly, a clinical investigation of hybrid ceramic fragment restorations with a 48-month follow-up demonstrated excellent adaptation and durability, further supporting their long-term clinical effectiveness [37]. In light of this evidence, together with their broad application in clinical practice, resin-matrix ceramics were chosen as the restorative material in the present study.

There is limited literature on the fracture resistance of resin-matrix ceramic onlays and crowns. Nonetheless, the present findings are consistent with prior research on other ceramics, which indicates that full crowns resist fracture better than overlays. One investigation examined crown length in three ceramic systems: glass ceramic (Dicor), feldspathic porcelain (Ceramco), and alumina-reinforced glass (In-Ceram) by testing occlusal crowns, half-length crowns, and full crowns until fracture. Results demonstrated superior resistance for full crowns (Ceramco: 963 N, Dicor: 852 N, In-Ceram: 1965 N) compared with occlusal (Ceramco: 478 N, Dicor: 633 N, In-Ceram: 1130 N) and half-length crowns (Ceramco: 667 N, Dicor: 700 N, In-Ceram: 1629 N) [38]. Another study tested CAD/CAM lithium disilicate overlays with margins at 2 mm and 4 mm above the gingiva and full crowns with gingival margins on maxillary second premolars. After 2,000,000 cycles, full crowns recorded the highest resistance (1018.8 N), followed by 2 mm overlays (813.8 N) and 4 mm overlays (436.1 N). The conclusion was that margin position plays a significant role, with crowns outperforming overlays [39].

Partial restorations in posterior teeth are increasingly used because they preserve more natural structure. Reports often describe them under different names, such as onlays or occlusal veneers [40,41,42]. In a clinical study of 27 cracked teeth restored with occlusal veneers and monitored for an average of 22.4 months, survival was 100% at one year and 92.6% at final follow-up, supporting their viability [43]. Furthermore, a systematic review and meta-analysis comparing onlays and partial crowns with full crowns for posterior teeth analyzed 4257 articles, selecting one randomized clinical trial and five observational studies. Results showed no significant differences in fracture rates, indicating that partial restorations can perform comparably to full crowns in the short term [44]. For this reason, partial restorations were evaluated in the present study.

The restorations in the present study were cemented onto 3D-printed resin dies rather than natural teeth, which may be considered by some as less clinically representative. However, several factors support the validity and advantages of this methodology for in vitro research. First, the dental resin used for the dies in this study has a tensile strength of 61 MPa, which lies within the range reported for natural dentin (44 to 97.8 MPa) [23,24]. This similarity in mechanical behavior suggests that the printed resin can serve as a reasonably comparable substrate to dentin for mechanical testing. In addition, the use of 3D-printed dies offers important methodological benefits, particularly in terms of standardization and reproducibility. Unlike natural teeth, which present considerable variations in anatomy, size, age, mineralization, and previous structural changes, printed dies allow the production of highly uniform specimens with consistent dimensions and properties. This reduces inter-sample variability and improves the reliability of comparisons among experimental groups. Furthermore, the use of resin dies is well documented in the literature, and similar 3D-printed or resin-based dies have been employed in previous in vitro studies evaluating the fracture resistance of ceramic restorations, further supporting the acceptability of this experimental model [45,46].

Further evidence supporting the use of resin dies is provided by a recent study that specifically investigated the effect of natural teeth and different die materials on the fracture resistance of zirconia crowns. In that investigation, multiple substrates were assessed, including three different brands of 3D-printed resin, one milled resin material, and natural teeth as the control group. Following adhesive cementation, the crowns were subjected to compressive loading until catastrophic failure occurred. The results demonstrated no statistically significant differences in fracture resistance between any of the resin-die groups and the natural-tooth group, indicating that resin printed dies can mimic the mechanical behavior of natural teeth under these testing conditions. Based on these findings, the authors concluded that resin dies constitute a viable and reliable alternative for crown cementation and fracture-load testing [47]. This observation is particularly important because it suggests that, when appropriate materials and protocols are used, printed resin dies can simulate the behavior of natural teeth with sufficient accuracy for controlled in vitro investigations, while simultaneously improving specimen standardization and experimental reproducibility.

From a methodological standpoint, the use of printed dies offers significant practical and scientific advantages. A major strength of this approach is the greater degree of standardization it provides, thereby reducing specimen-to-specimen variability. This is particularly relevant in fracture-resistance studies, in which subtle differences in preparation geometry, dimensions, or substrate properties may influence the final outcomes. The fabrication of printed dies with uniform morphology and consistent material characteristics allows for better control of experimental conditions and supports more reliable comparisons among study groups. Moreover, printed dies help address several logistical and methodological limitations associated with extracted human teeth. The collection of an adequate number of sound, caries-free, and anatomically similar teeth is often difficult, and the preparation of multiple natural teeth in an identical manner is complicated by inherent biological variability. In addition, natural teeth may be affected by dehydration, storage conditions, or structural changes during specimen handling and preparation, which may alter their mechanical response. By reducing these confounding factors, printed dies enhance reproducibility, improve standardization, and strengthen the internal validity of the study. Therefore, when considering their comparable material properties, the evidence available in the literature, and the methodological benefits of a more controlled and standardized protocol, the use of 3D-printed resin dies in this in vitro investigation can be regarded as a justified and appropriate experimental choice.

Although the results of the present study demonstrated that full-coverage crowns provided higher fracture resistance than all overlay restorations, the fracture resistance values recorded for the overlay groups should still be considered clinically favorable. Notably, even the group with the lowest value (OvF.4m), at 1257 N, exhibited fracture resistance substantially greater than the occlusal forces reported for premolar teeth in normal function, which are approximately 300 N for the first premolar and 450 N for the second premolar [48]. Furthermore, these values exceeded those reported for individuals with higher occlusal loads and parafunctional habits, such as bruxism, in whom bite forces are estimated to reach around 700 N [49,50]. Based on these findings, overlay restorations may be regarded as a clinically acceptable option. In addition to offering a more conservative treatment approach by preserving more tooth structure than conventional full-coverage crowns, they also demonstrate the capacity to resist occlusal forces well beyond those typically encountered under functional and many.

Furthermore, the overlay designs presented in this study have margins located above the gingival level, which may provide several important biological and clinical advantages. Supragingival margins are generally considered more favorable for periodontal health because they are not placed within the gingival sulcus, thereby reducing the likelihood of violating the biologic width. As a result, they may help minimize soft tissue irritation and decrease the risk of gingival inflammation, recession, pocket formation, and long-term periodontal breakdown. From a restorative perspective, supragingival margins also simplify the clinical procedure because they are fully visible and more accessible to the clinician, making them easier to prepare, refine, and finish accurately with rotary instruments. Their visibility also facilitates more precise impression making or digital scanning, which may contribute to improved marginal adaptation of the final restoration. In addition, supragingival margins reduce the risk of cement-related complications, since excess luting material can be more easily detected and thoroughly removed after cementation. Another important benefit is that these margins are more favorable for patient maintenance, as they are easier to clean during daily oral hygiene procedures, thereby reducing plaque retention and helping to maintain long-term periodontal stability. Therefore, in addition to preserving tooth structure, overlay restorations with supragingival margins may offer meaningful advantages in terms of periodontal preservation, clinical handling, restorative accuracy, and long-term maintenance [51,52].

In the present study, the cemented restorations were also subjected to artificial aging through thermocycling (10,000 cycles, 5–55 °C) before fracture testing. This aging protocol was selected to simulate the thermal stresses that restorations experience in the oral environment as a result of repeated exposure to hot and cold temperatures. According to the literature, 10,000 thermocycles approximate roughly 1 year of clinical service, based on the commonly accepted estimate that 20 to 50 cycles correspond to 1 day of in vivo function [53,54]. Thus, the thermocycling regimen used in this study represents a meaningful attempt to reproduce the aging conditions that restorations may encounter during early clinical function. Moreover, this protocol is consistent with prior in vitro investigations, as several studies evaluating the fracture resistance of dental restorations have also used 10,000 cycles before mechanical testing [55,56]. The use of this established thermocycling regimen strengthens the comparability of the present findings with previously published data. Another limitation of the study is the lack of periodontal ligament simulation. Therefore, future studies should evaluate fracture resistance with periodontal ligament simulation, as this would allow for a more clinically realistic assessment of the interaction between restorations and the periodontal ligament.

## 5. Conclusions

This study confirms that margin location plays a significant role in the fracture resistance of restorations fabricated with chairside CAD/CAM resin-matrix ceramics. Conventional crowns showed the highest resistance, surpassing all overlay designs. Within the overlay groups, restorations with margins placed 2 mm above the gingival line showed greater resistance than those placed 4 mm above the gingival line. The lowest values were found in overlays combining a 4 mm supragingival margin with a flat occlusal surface. Overall, the findings emphasize the critical role of preparation design in restorations for maxillary premolars, underscoring that both margin position and occlusal morphology strongly influence performance.

## Figures and Tables

**Table 1:** Mean fracture resistance of CAD/CAM resin-matrix ceramic restorations with different preparation designs compared with full crowns in maxillary premolars.

Group	Type of Restoration	Number of Samples	Fracture Resistance (±SD), N	Range (Min–Max)
Group 1 (Ov.2m)	Overlay with margin 2 mm above gingival level	16	1605.69 (88) ^a^	1501–1791
Group 2 (Ov.4m)	Overlay with margin 4 mm above gingival level	16	1403.81 (63) ^b^	1310–1491
Group 3 (OvF.4m)	Overlay with margin 4 mm above gingival level and flat occlusal surface	16	1257.38 (73) ^c^	1122–1353
Group 4 (FCC)	Full crown with margin at gingival level	16	1838.00 (106) ^d^	1669–2091

Note. Different superscript letters indicate statistically significant differences (Tukey HSD, α = 0.05). Abbreviations: SD = standard deviation; N = Newtons.

## Data Availability

Data presented in this study are available on request from the corresponding authors.
